# Memory-Efficient Searching of Gas-Chromatography Mass Spectra Accelerated by Prescreening

**DOI:** 10.3390/metabo12060491

**Published:** 2022-05-29

**Authors:** Aleksandr Smirnov, Yunfei Liao, Xiuxia Du

**Affiliations:** Department of Bioinformatics and Genomics, College of Computing and Informatics, University of North Carolina at Charlotte, Charlotte, NC 28223, USA; asmirno1@uncc.edu (A.S.); yliao13@uncc.edu (Y.L.)

**Keywords:** metabolomics, compound annotation, library search

## Abstract

The number of metabolomics studies and spectral libraries for compound annotation (i.e., assigning possible compound identities to a fragmentation spectrum) has been growing steadily in recent years. Accompanying this growth is the number of mass spectra available for searching through those libraries. As the size of spectral libraries grows, accurate and fast compound annotation becomes more challenging. We herein report a prescreening algorithm that was developed to address the speed of spectral search under the constraint of low memory requirements. This prescreening has been incorporated into the Automated Data Analysis Pipeline Spectral Knowledgebase (ADAP-KDB) and can be applied to compound annotation by searching other spectral libraries as well. Performance of the prescreening algorithm was evaluated for different sets of parameters and compared to the original ADAP-KDB spectral search and the MSSearch software. The comparison has demonstrated that the new algorithm is about four-times faster than the original when searching for low-resolution mass spectra, and about as fast as the original when searching for high-resolution mass spectra. However, the new algorithm is still slower than MSSearch due to the relational database design of the former. The new search workflow can be tried out at the ADAP-KDB web portal.

## 1. Introduction

Comprehensive fragmentation mass spectral libraries are essential for interpreting spectra derived from raw untargeted gas chromatography coupled to mass spectrometry (GC-MS) data. This interpretation is carried out by matching query mass spectra to a library of reference mass spectra of known and, sometimes, unknown compounds. Examples of such libraries include the National Institute of Standards and Technology (NIST) EI-MS Library with more than 300 k GC-MS spectra of known compounds [1], MassBank of North America with 200 k+ mass spectra from experimental and in silico libraries [2], and Automated Data Analysis Pipeline Spectral Knowledgebase (ADAP-KDB) with its number of mass spectra of both known and unknown compounds constantly increasing [3].

Evidently, present-day mass spectral libraries contain hundreds of thousands of mass spectra. However, the evergrowing number of spectra in these libraries raises several challenges for the library matching. First, the increase of the total number of mass spectra in a library may lead to the increased false-positive matching rate. In other words, a huge library size increases the possibility that the correct spectrum hit will be buried among incorrect hits with similar matching scores. This first challenge is usually addressed by improving methods of calculating the spectral similarity score [4,5].

The second challenge is a result of the high time complexity of calculating the similarity scores between a query spectrum and thousands of library spectra. Considering the fact that untargeted mass spectrometry data from a single study could contain hundreds of mass spectra, it can easily take hours to match all query spectra to all library spectra because calculating spectral similarity scores is very time-consuming. This challenge is resolved by introducing two stages of the library matching. The first stage (prescreening search) uses a fast algorithm to find candidate mass spectra that may produce a high similarity score when compared to the query spectrum. Then, the second stage (main search) actually calculates those similarity scores. This two-stage approach helps to significantly accelerate the library matching if the prescreening search is fast, returns a small number of candidate spectra, and those candidate spectra contain the correct hit.

Since the earliest days of creating mass spectral libraries, researchers worked on developing algorithms for fast library matching [6]. Most of these algorithms are based on selecting a small subset of spectral peaks and then comparing those selected peaks between the query and library spectra. For instance, there were proposed techniques of selecting *n* most intense peaks in a spectrum [7] or selecting *n* most intense peaks in each pre-defined m/z interval [6,8]. Similar techniques are applied in the NIST MSSearch to perform the prescreening spectral search, where the eight most intense peaks in the query spectrum are compared to the sixteen most intense peaks in the library spectra [9]. Finally, several recent developments suggest performing prescreening by comparing highest-intensity peaks combined with highest-mass peaks [10,11] or low-frequency coefficients of the discrete wavelet transform of each spectrum [12]. In the most recent years, there appeared a number of deep learning [13,14,15] and hash-based [16] methods that construct new time-efficient similarity scores that improve identification of a compound or at least its chemical structure. However, their efficiency and applicability to different types of mass spectra is still under study [15].

When the ADAP-KDB Spectral Knowledgebase was first created, a naive spectral search approach was implemented by storing all library mass spectra in a relational database and executing Structured Query Language (SQL) queries to calculate similarity scores between those spectra and the spectra uploaded by a user [3]. This approach had benefits of (i) a seamless integration between a spectral database and the ADAP-KDB web server; (ii) easy management and dynamic update of the relationships between users, their accounts, and their spectral libraries; (iii) low CPU and memory requirements; and (iv) database indexing mechanisms for a fast search. However, the original ADAP-KDB spectral search was essentially calculating the similarity score between each user spectrum and each library spectrum in ADAP-KDB, which was still very slow. As a result, it quickly became evident that the speed of the ADAP-KDB spectral search needed to be improved. In order to hasten the ADAP spectral search, the two-stage approach was adapted with a quick prescreening of all mass spectra in a library and a relatively slower calculation of the spectral similarity scores between a user spectrum and the library spectra selected by the prescreening.

Despite the large number of proposed techniques to perform the prescreening and main spectral search, NIST MSSearch and its search algorithms continue to be among the most recognized and reliable. Therefore, the NIST MSSearch prescreening technique was chosen as the basis for our investigation of the optimal parameters for the prescreening to be used in ADAP-KDB and quantitative evaluation of the resulting improvement in search speed. In this investigation of the prescreening, the top *n* most intense peaks in a query spectrum are compared to the top *m* most intense peaks in the library spectra. Then, all matching library spectra are ranked based on the number of peaks they have in common with the query spectrum. Finally, only a small number (controlled by parameter *R*) of the top-ranked library spectra are returned from the prescreening for calculating the spectral similarity score between them and the query spectrum. It is hoped that this investigation could shed light on the utility of the prescreening and broaden the adoption of this approach in the search algorithm in other spectral libraries as well.

In the following sections, we describe the prescreening search technique implemented in ADAP-KDB, the systematic investigation for determining the optimal combination of prescreening parameters {n,m,andR}, and evaluation of the performance and applicability of the prescreening to both low-mass-resolution and high-mass-resolution spectra.

## 2. Results

### 2.1. Original ADAP-KDB Spectral Search Algorithm

ADAP-KDB Spectral Knowledgebase [3] was first created for tracking and prioritizing unknown compounds across Metabolomics studies in the NIH’s Metabolomics Data Repository (NMDR). Specifically, ADAP-KDB provided the search functionality through a publicly available library of consensus mass spectra of both known and unknown compounds. Those consensus mass spectra were constructed after processing raw untargeted MS data of the studies publicly available through NMDR, and clustering all mass spectra derived from those studies. As a result, ADAP-KDB users could search the library of consensus spectra, view the corresponding constituent spectra, and link them to the studies those spectra come from. In addition to searching against the consensus spectra, the functionality of ADAP-KDB was soon expanded to allow users to upload their own private spectral libraries and search against them. The latter increased the versatility of ADAP-KDB and made it useful for matching against various in-house libraries.

From its initial implementation, ADAP-KDB has been using the general-purpose relational database MySQL [17] to store spectral data. Although not the fastest in terms of the pure speed of spectral search, this solution was a good fit for a web server with low system requirements (2 CPUs, 16 GB RAM) hosted on Amazon Web Services [18]. Using a general-purpose relational database simplified the development process and had several advantages. First, a relational database inherently provides support for adding, updating, and deleting spectral library data, while maintaining its integrity and concurrency. Second, relational databases are memory-efficient and do not require much RAM to operate, which makes them well-suited for cost-effective web applications. Finally, database indexing can accelerate certain data operations such as matching a query m/z value to all spectral m/z values in the database.

The original ADAP-KDB spectral search algorithm calculated the similarity between a query spectrum and a library spectrum as the sqrt-cosine similarity of peak intensities between them after the peak intensities have been scaled and then transformed [3]. The sqrt-cosine similarity between library spectrum *L* and user spectrum *U* is calculated by the formula:

(1) $$S\left(U,L\right)=\frac{{\left(\sum_{i}\sqrt{A_{i}^{u}\;\cdot \;A_{i}^{l}}\right)}^{2}}{\sum_{i}A_{i}^{u}\;\cdot \;\sum_{i}A_{i}^{l}}$$

Here, $A_{i}^{u}$ and $A_{i}^{l}$ are the scaled intensities of peaks in the spectra *U* and *L*, respectively, and the summations are taken over all m/z values in the corresponding spectra. Finally, the peak intensity is scaled as follows [19]:

(2) $$A_{i}=I_{i}\;\cdot \;mz_{i}/\left(1+\omega I_{i}\right)$$

where $I_{i}$ and $mz_{i}$ are the original intensity and m/z-value of the *i*-th peak in a spectrum (assuming the base peak intensity equals 1), ω=1/(I−0.5) is a weighting factor designed to apply a correction to the spectra with a single dominant peak, and $I=\sum_{i}I_{i}$ is the total intensity of the spectrum.

In order to quickly compute the spectral similarity in Equation (1) between a query spectrum and all of the library spectra, the similarity score is calculated directly by the relational database containing the spectral information. Listing 1 shows a pseudo SQL query to calculate the similarity score in Equation (1). The spectral information of the library spectra is stored in the Table Peak with columns Mz, Intensity, TotalIntensity, and SpectrumId, while the query spectrum is represented by the m/z-intensity pairs $\left(mz_{1},A_{1}^{u}\right),\ldots ,\left(mz_{n},A_{n}^{u}\right)$. Lines 2–3 in the SQL query return library spectrum IDs and the product $\sqrt{A_{i}^{l}\;\cdot \;A_{1}^{u}}$ for scaled intensities $A_{i}^{l}$ of all library peaks with their m/z values close to $mz_{1}$ in the query spectrum. The same operations are executed for the pairs $\left(mz_{2},A_{2}^{u}\right),\left(mz_{3},A_{3}^{u}\right),\ldots ,\left(mz_{n},A_{n}^{u}\right)$ of the query spectrum. Finally, lines 1 through 10 return ID and the spectrum similarity score (1) for each library spectrum if that score exceeds the spectral similarity score threshold. Thus, the pseudo SQL query returns IDs of the library spectra and the corresponding similarity scores.

**Listing 1.** A pseudo SQL query for calculating similarity scores between a query spectrum and all library spectra. The spectral information for all library spectra is stored in Table Peak with columns Mz, Intensity, TotalIntensity, and SpectrumId. The spectral information for the query spectrum is represented by the m/z values $mz_{1},\ldots ,mz_{n}$ and the corresponding scaled intensities $A_{1}^{u},\ldots ,A_{n}^{u}$ and the total scaled intensity $A^{u}=\sum_{i=1}^{n}A_{i}^{u}$. This pseudo SQL query returns a table with similarity scores and IDs of the library spectra with scores greater than the spectral similarity score threshold.

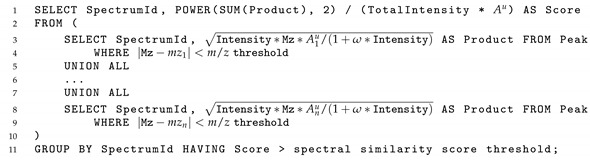



This original ADAP-KDB spectral search algorithm takes advantage of a database indexing algorithm applied to the Mz column of the Peak table to provide a fast look-up of matching m/z values in the library spectra. Moreover, it avoids transferring large amounts of data between a database and a web server, which is generally one of the slowest bottlenecks of computational systems. As a result, it is much faster than retrieving every spectrum from the database and calculating the spectral similarity score on the web server. However, as the number of spectra in ADAP-KDB Spectral Knowledgebase grows, the spectral search algorithm would take an increasing amount of time to calculate spectral similarities. Thus, a faster approach for performing the library search was needed.

### 2.2. New ADAP-KDB Spectral Search Algorithm with the Prescreening Search

Speed of the spectral search in ADAP-KDB has been significantly improved by implementing the prescreening search step. The purpose of the prescreening search is to quickly find candidate spectra so that similarity scores are calculated only between the query spectrum and those candidate spectra. Such a prescreening search can drastically improve the speed of the spectral search if the prescreening satisfies the following conditions: (i) prescreening algorithm is much faster than the main search algorithm; (ii) prescreening algorithm returns a relatively small number of candidate spectra; and (iii) the returned candidate spectra contain the correct match to the query spectrum.

**Figure 1:**
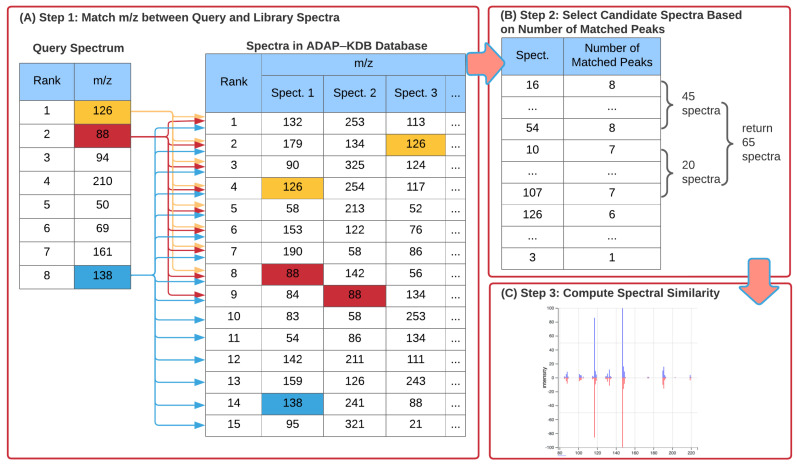
New ADAP-KDB spectral search algorithm with the prescreening search: (**A**) m/z values of 8 largest peaks in the query spectrum are matched to m/z values of 15 largest peaks in every library spectrum; (**B**) all library spectra are ranked based on the number of matched m/z values, and top 50+ candidate spectra are returned by the prescreening search; (**C**) the similarity score is calculated for each candidate spectrum.

Figure 1 shows the three steps of a spectral search algorithm with the prescreening search. First, m/z values of the largest peaks in all library and query spectra are pre-calculated. Then, the m/z value of the largest peak in the query spectrum is matched to m/z values of *n* largest peaks in the library spectra; m/z value of the second largest peak in the query spectrum is matched to m/z values of n+1 largest peaks in the library spectra; and so forth until m/z value of the *n*-th largest peak in the query spectrum is matched to m/z values of *m* largest peaks in the library spectra (Figure 1A). After the peaks are matched, the library spectra are ranked by the number of matched peaks (Figure 1B). Next, the candidate spectra are determined as follows: all library spectra with *n* matched peaks are included into the list of candidate spectra; if the number of those spectra is below threshold *R*, then all library spectra with n−1 matched peaks are included into the candidate spectra; if the total number of candidate spectra is still below threshold *R*, then all library spectra with n−2 matched peaks are included into the candidate spectra. This process continues until either the number of candidate spectra exceeds the threshold *R* or all library spectra with at least one matched peak have been included into candidate spectra. Finally, spectral similarities between the query spectrum and the candidate spectra are calculated, and the candidate spectra with the highest scores are returned to the user (Figure 1C).

To truly accelerate the overall library search, this prescreening search algorithm should be significantly faster than calculating the spectral similarity score for every library spectrum as long as parameters *n* and *m* are small enough. At the same time, parameters *n*, *m*, and *R* should be large enough to ensure that candidate spectra returned by the prescreening contain the correct library matches. In order to determine the optimal values of these parameters, we investigated the performance of the prescreening algorithm with different sets of parameters on both low-mass-resolution and high-mass-resolution spectra.

### 2.3. Comparison of ADAP-KDB Spectral Search Algorithms

Performance of the prescreening search algorithms was evaluated by searching 1111 mass spectra acquired at the low-mass resolution and 1447 mass spectra acquired at the high-mass resolution. These mass spectra were obtained after preprocessing freely-available raw untargeted GC-MS metabolomics data for studies ST000381 and ST001056 from the Metabolomics Workbench [20]. The preprocessing was performed by the ADAP-GC workflow [21]. Each mass spectrum was matched against the ADAP-KDB Spectral Knowledgebase containing about 20,000 low-resolution and high-resolution GC-MS library spectra available for search. All spectral matchings were executed on a local machine with 2.9 GHz Intel Core i5 CPU, 16 GB RAM, and macOS Big Sur operating system. The spectral search was performed first by the original ADAP-KDB algorithm and then by the new algorithm with the prescreening search executed with different sets of parameters *n*, *m*, and *R*. For detailed information of each spectral search, see the Supporting Information.

Two performance characteristics were computed for each executed spectral search. First, the speed of the new algorithm was compared to that of the original ADAP-KDB spectral search by calculating their execution times for each query spectrum. When the original ADAP-KDB spectral search was used, the execution time averaged to 12.522 s for searching a single low-mass-resolution spectrum and 3.399 s for a high-mass-resolution spectrum. This observed difference in searching times between low-mass-resolution and high-mass-resolution spectra can be explained by significantly fewer number of spectral peaks in the latter. We calculated the average number of matching peaks with close m/z values during the search of low-mass-resolution and high-mass-resolution spectra. Indeed, when a mass spectrum at low resolution is compared against the ADAP-KDB library, the Peak table contains 27 matching peaks on average for each query m/z value. However, when a mass spectrum at high resolution is compared against ADAP-KDB, the average number of matched peaks from the Peak table is only 4.9 for each query m/z value (Table S5). Therefore, the number of rows returned by the lines 3–9 of the SQL query in the original algorithm (Figure 1) is about 5 times greater for a low-mass-resolution query spectrum than that for a high-mass-resolution. This explains the substantial differences in the search times between the spectra at low and high resolutions.

**Figure 2:**
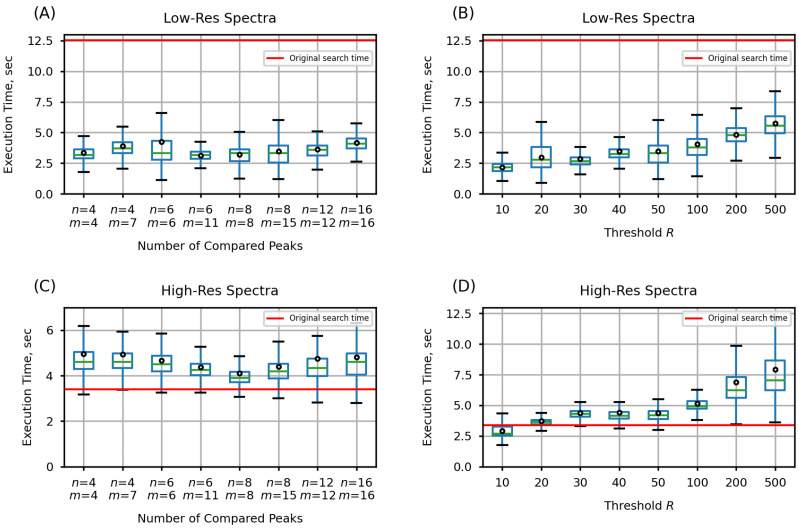
Execution times of the new search algorithm, estimated for prescreening parameters (n,m,R), where *n* is the number of query spectrum peaks participating in the prescreening search, *m* the number of library spectrum peaks participating in the prescreening search, and *R* determines the number of candidate spectra returned by the prescreening search: (**A**,**B**) search against low-resolution spectra; (**C**,**D**) search against high-resolution spectra.

After adding the prescreening search to ADAP-KDB, the average execution time has decreased down to 2–8 s for low-resolution spectra, depending on the chosen parameters (Figure 2). Specifically, the number of the largest peaks participating in the prescreening search have demonstrated almost no influence on the execution time of the search algorithm (Figure 2A,C). However, when the parameter *R* was varied from 10 to 500, it affected the number of candidate spectra returned by the prescreening search, which resulted in an increase of the search execution times from about 2 s to 6 s for the low-resolution spectral search and 8 s for the high-resolution spectral search (Figure 2B,D). However, even with R=500, the new algorithm is still much faster when searching against spectra acquired at the low-mass resolution.

**Figure 3:**
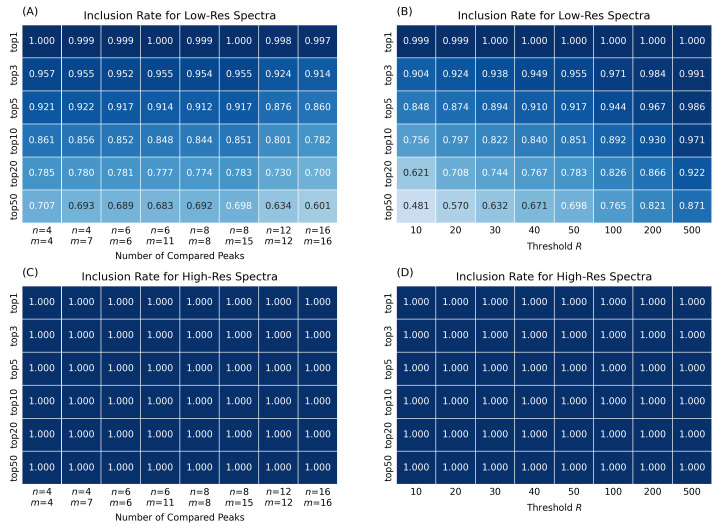
Inclusion rates of the new search algorithm, estimated for prescreening parameters (n,m,R), where *n* is the number of query spectrum peaks participating in the prescreening search, *m* the number of library spectrum peaks participating in the prescreening search, and *R* determines the number of candidate spectra returned by the prescreening search: (**A**,**B**) search against low-resolution spectra; (**C**,**D**) search against high-resolution spectra.

The second evaluated performance characteristic is the average inclusion rate, which estimates what fraction of the top-*N* spectral matches with a matching score (1) greater than 0.6 returned by the original ADAP-KDB spectral search was retained after adding the prescreening search (see Equation (3)). First, the inclusion rate of the top-1 match is very close to 1.0 regardless of what parameters *n*, *m*, and *R* are used (Figure 3). This indicates that the best spectral match is almost always included in the set of candidate library spectra returned from the prescreening. The inclusion rate of the top-3 matches is above 0.9 for the low-mass-resolution spectra (Figure 3A). In general, the inclusion rate decreases for the low-resolution searches as more library matches for one query spectrum are expected to be retained. For instance, the inclusion rate of the top-50 matches shows that only around half of the spectral matches are retained by the prescreening search. These results show that the prescreening search tends to retain the top-scoring spectral matches and reject low-ranked matches for the low-mass-resolution spectra, demonstrating the advantages of the prescreening in terms of reducing possible false compound annotations. As for the inclusion rate of the prescreening searches for high-mass-resolution spectra, it stays at 1.0 for any sets of parameters *n*, *m*, and *R* (Figure 3C,D). This can be explained by the fact that high-mass-resolution spectra have smaller number of peaks, thus 4+ largest peaks are sufficient to find all matching library spectra.

It is interesting to consider the inclusion rate for different prescreening search strategies. Most prescreening algorithms compare the same number of peaks in both the query and library spectra [6,12]. In our tests, this approach is represented by five pairs of parameters when both the number of compared peaks in the query spectrum *n* and the number of compared peaks in the library spectrum *m* are set to 4, 6, 8, 12, and 16. In addition, the NIST MSSearch User Guide [9] describes a slightly different approach with eight largest peaks in the query spectrum being compared to fifteen largest peaks in the library spectra. This NIST MSSearch approach is further investigated in our work by considering three pairs of parameters: (n=4,m=7), (n=6,m=11), and (n=8,m=15). Although the latter approach is slower, its execution takes less than a second longer than the former approach (Figure 2A). At the same time, the NIST MSSearch approach shows consistently better inclusion rate for top-1 through top-10 matches (Figure 3A). Therefore, the MSSearch approach of the prescreening search performs better than comparing the same number of peaks in both query and library spectra.

## 3. Discussion

Overall, the comparison of the original ADAP-KDB spectral search and the new algorithm with the prescreening search shows that performance of the library search with prescreening is about four-times faster for the low-mass-resolution spectra and very similar for the high-mass-resolution spectra. Moreover, the execution time stays about the same for all pairs of parameters *n* and *m*, while pairs (n=4,m=7), (n=6,m=11), and (n=8,m=15) demonstrate slightly better inclusion rate than pairs (n=4,m=4), (n=6,m=6), and (n=8,m=8), respectively. Based on these results, comparing eight largest peaks in the query spectrum to 15 largest peaks in the library spectra seem to be the optimal approach for the preliminary search. Selecting threshold *R* is based on the tradeoff between the execution time and the inclusion rate. Based on the comparison results, value R=50 seems to be optimal for keeping high inclusion rate and low execution time for both low-mass-resolution and high-mass-resolution spectra. Therefore, these values of the parameters were selected as optimal for the prescreening search of the new spectral search algorithm. The latter algorithm has been incorporated into ADAP-KDB and can be tested by visiting the ADAP-KDB web portal at www.adap.cloud (accessed on 21 February 2022).

Table 1 shows the average execution time (in seconds) of searching both low-resolution and high-resolution spectra in ADAP-KDB and NIST MSSearch. Because ADAP-KDB is run on Linux/Mac, while NIST MSSearch requires the Windows operating system, we had to use a different machine (2.5 GHz Intel Xeon (R), 98 GB RAM, Windows Server 2016) for the latter. The ADAP-KDB library spectra were exported into MSP files and then converted into the NIST MSSearch library format as an EI library (for using with the Identity search) and a high-resolution library (for using with the In-source HighRes search). The comparison shows that even though the new ADAP-KDB search is much faster than the original, NIST MSSearch is still faster than ADAP-KDB for both high-mass-resolution and low-mass-resolution spectra. However, when considering the performance differences between ADAP-KDB and NIST MSSearch, one should remember that NIST MSSearch is a Windows-native local application, while ADAP-KDB is a web application with multi-user support and low system requirements. As such, the performance of ADAP-KDB is comparable to that of NIST MSSearch, and we expect it to scale in a similar way.

Thus far, we have evaluated and implemented the prescreening-aided spectral search approach based on the NIST MSSearch prescreening algorithm. However, a number of other search approaches have been proposed in recent years. As the number of spectra in ADAP-KDB grows, we will continuously improve our search algorithm to meet the growing needs of fast spectral search in ADAP-KDB and other spectral libraries.

## 4. Materials and Methods

Publicly available raw untargeted GC-MS data for studies ST000381 (low mass resolution) and ST001056 (high mass resolution) were downloaded from the NMDR [20]. The raw data was processed with the ADAP-GC computational workflow [21], which consists of 5 steps: chromatogram building, peak detection, spectral deconvolution, alignment, and ANOVA significance test. Parameters of each preprocessing step are listed in Table S6. The produced mass spectra were exported into two MSP files.

The spectral search was executed in ADAP-KDB Spectral Knowledgebase [3], deployed locally on a workstation with 2.9 GHz Intel Core i5 CPU, 16 GB RAM, and macOS BigSur operating system. The code of ADAP-KDB was modified to output timestamps that were used to calculate execution times of the original search algorithm and the new search algorithm with prescreening. The inclusion rate between the top-N original and new search results for each study is calculated as follows:

(3) $$Inclusion\;Rate\left(N\right)=\frac{M_{pre}\left(N\right)}{M_{orig}\left(N\right)}$$

where $M_{orig}\left(N\right)$ is the total number of top-N matches with spectral similarity above 0.6 returned by the original algorithm across all query spectra in an MSP file, $M_{pre}\left(N\right)$ is the number of the same matches returned by the algorithm with prescreening.

In order to compare the ADAP-KDB spectral search to NIST MSSearch, ADAP-KDB high-resolution and low-resolution GC-MS libraries were exported into MSP files and converted into the proprietary NIST MSSearch format. Then, the library matching was performed by NIST MSSearch on a workstation with 2.5 GHz Intel Xeon (R) CPU, 98 GB RAM, and Windows OS.

## 5. Conclusions

A new memory-efficient algorithm with prescreening was implemented for searching GC-MS spectra in ADAP-KDB Spectral Knowledgebase. Its performance was evaluated by comparing to both the original ADAP-KDB spectral search algorithm and the NIST MSSearch software. The evaluation demonstrated that the prescreening greatly improves the speed of the spectral search for the mass spectra acquired at the low-mass resolution. For those spectra, the new algorithm is about four-times faster than the original ADAP-KDB spectral search and retains a majority of the top-ranked hits. However, when a search is performed for the spectra acquired at the high-mass resolution, performances of the new and original approaches are very similar both in speed and the number of hits. The source code for ADAP-KDB is publicly available through GitHub at https://github.com/du-lab/ADAP-compound-DB (accessed on 21 February 2022).

## Figures and Tables

**Table 1:** Average execution time of each spectral search per spectrum (in seconds). The columns (from left to right) correspond to the original ADAP-KDB spectral search, new ADAP-KDB spectral search with prescreening, the NIST MSSearch identity search for low-resolution spectra, the NIST MSSearch in-source HighRes similarity search.

	ADAP-KDB (Orig)	ADAP-KDB (Pre)	MSSearch (Id)	MSSearch (High-Res)
1111 low-res spectra	12.5	3.5	1.1	—
1446 high-res spectra	3.4	4.4	1.2	1.0

## Data Availability

The data presented in this study are openly available in the NIH Common Fund’s National Metabolomics Data Repository (NMDR) website, the Metabolomics Workbench, https://www.metabolomicsworkbench.org where it has been assigned Project IDs PR000299 and PR000709. The data can be accessed directly via Project DOI: 10.21228/M8D60P and 10.21228/M81M4X respectively. This work is supported by NIH grant U2C-DK119886.

## Supplementary Materials

The following are available online at https://www.mdpi.com/article/10.3390/metabo12060491/s1, Tables S1–S4: Average running times and inclusion rates for low-resolution and high-resolution spectra and each combination of parameters (*m*, *n*, *R*), Table S5: Average number of matched peaks for low-resolution and high-resolution spectra, Figure S1: Comparison between calculating inclusion rates for low-resolution and high-resolution spectra, Figure S2: Pseudo SQL code of the ADAP-KDB prescreening algorithm, Table S6: ADAP-GC 4.0 Data Processing Parameters.
