# Supplementary material for: Memory-Efficient Searching of Gas-Chromatography Mass Spectra Accelerated by Prescreening
Source: Metabolites. 2022 May 29;12(6):491. doi: 10.3390/metabo12060491 (PMC9229217; doi:10.3390/metabo12060491)
Supplement: Supplementary file 1 [file metabolites-12-00491-s001.zip › metabolites-1629762-supplementary.pdf]

Supplementary Materials: Preliminary search algorithm for fast matching of mass spectra in ADAP-KDB spectral knowledgebase

Aleksandr Smirnov [1,†], Yunfei Liao [1,†] and Xiuxia Du [1*]

1. Comparison of the search algorithms with and without pre-screening for the low-resolution data

Table S1: Running time for low resolution data

Prescreen parameters			Average Runing Time (sec)
R	*n*	*m*	
50	4	4	3.342
	4	7	3.888
	6	6	4.236
	6	11	3.135
	8	8	3.202
	8	15	3.495
	12	12	3.648
	16	16	4.189
10	8	15	2.160
20			2.982
30			2.852
40			3.487
50			3.495
100			4.047
200			4.842
500			5.760
The original algorithm			12.522

Table S1 presents the running time of using 1110 low-resolution spectra to search against ADAP-KDB library spectra with different preliminary search parameters and the running time of the original algorithm without the preliminary search. In Table S1, n is the number of most intense peaks in the query spectrum, m is the number of most intense peaks in the library spectrum that participate in the preliminary search. R is the minimum number of library spectra returned as candidate spectra for calculating similarities with query spectrum.

Table S1 indicates that the average running time of using one user's spectrum to search against ADAP-KDB library spectra is around 3 seconds when the threshold (R) is 50. In addition, when the preliminary search parameter is using 8 peaks (n=8) of the user's spectrum to compare with 15 peaks (m=15) of the ADAP-KDB library spectra, the average running time of using one user's spectrum to search against ADAP-KDB library spectra increases from 1.783 seconds to 4.797 seconds when R is raised from 10 to 500. Table S1 also shows that the average time cost of the original search algorithm is 42.442 seconds, i.e. the search algorithm with pre-screening is about 10 times faster than the original ADAP-KDB search algorithm.

Table S2: Inclusion rate for low resolution data

Prescreen Parameters			Inclusion Rate					
R	*n*	*m*	Top 1	Top 3	Top 5	Top 10	Top 20	Top 50
50	4	4	1	0.957	0.921	0.861	0.785	0.707
	4	7	0.999	0.955	0.922	0.856	0.780	0.693
	6	6	0.999	0.952	0.917	0.852	0.781	0.689
	6	11	1	0.955	0.914	0.848	0.777	0.683
	8	8	0.999	0.954	0.912	0.844	0.774	0.692
	8	15	1	0.955	0.917	0.851	0.783	0.698
	12	12	0.998	0.924	0.876	0.801	0.730	0.634
	16	16	0.997	0.914	0.860	0.782	0.700	0.601
10	8	15	0.999	0.904	0.848	0.756	0.621	0.481
20			0.999	0.924	0.874	0.797	0.708	0.570
30			1	0.938	0.894	0.822	0.744	0.632
40			1	0.949	0.910	0.840	0.767	0.671
50			1	0.955	0.917	0.851	0.783	0.698
100			1	0.971	0.944	0.892	0.826	0.765
200			1	0.984	0.967	0.930	0.866	0.821
500			1	0.991	0.986	0.971	0.922	0.871

Table S2 displays the inclusion rates between the matched spectra obtained by the original and preliminary search algorithms containing the different parameters.

The inclusion rate is calculated following the three steps. Firstly, the user uploads their .msp file to search against the ADAP-KDB library with the original search algorithm. After this, a list of matched spectra (List A) for each query spectrum with the corresponding similarity score will be achieved (List A only contains the matched spectra which matching scores are greater than 0.6). The list of library spectra is ranked by the similarity score and ordered by both query spectra and ranking. Secondly, use the same .msp file against ADAP-KDB with the pre-screening algorithm and obtain a new list of matched spectra (List B) for each query spectrum with the corresponding similarity scores. For example, the Top1 inclusion rate is calculated by counting how many matched spectra labeled rank 1 in List A are presented in List B. If there are three Top1 matches in List A, and all of them are presented in List B, then the inclusion rate is 3/3 = 1. If only two are presented in the pre-screening algorithm, then the inclusion rate is 2/3.

From Table S2, the matched spectra with the highest rank (Top 1), containing all listed parameters, had inclusion rates close to or equal to 1. The inclusion rates decrease when the rank of matched spectra increases and at the 20th ranking (Top 20), the inclusion rates drop significantly to about 0.5.

Overall, compared to the original search algorithm, the average running time of using one user's spectrum to search against ADAP-KDB library spectra has improved more than 10 times with the preliminary search algorithm. Moreover, the inclusion rates between the original search algorithm and the preliminary search algorithm containing all listed parameters are close to 1 at Top 1 rank and close to 0.9 at Top 3 rank.

2. Comparison of the search algorithms with and without pre-screening for the high resolution data

Table S3: Running time for high resolution data

Prescreen parameters			Average Runing Time (sec)
R	*n*	*m*	
50	4	4	4.960
	4	7	4.926
	6	6	4.668
	6	11	4.368
	8	8	4.108
	8	15	4.398
	12	12	4.738
	16	16	4.815
10	8	15	2.935
20			3.714
30			4.368
40			4.432
50			4.398
100			5.167
200			6.890
500			7.935
The original algorithm			3.399

Table S3 displays the results of running time when using 1447 high-resolution spectra to search against ADAP-KDB library spectra with the same preliminary search parameters listed in Table S1. The running time of using the original search algorithm is also included in Table S3.

Table S4: Inclusion rate for high resolution data

Prescreen Parameters			Inclusion Rate					
R	*n*	*m*	Top 1	Top 3	Top 5	Top 10	Top 20	Top 50
50	4	4	1	1	1	1	1	1
	4	7	1	1	1	1	1	1
	6	6	1	1	1	1	1	1
	6	11	1	1	1	1	1	1
	8	8	1	1	1	1	1	1
	8	15	1	1	1	1	1	1
	12	12	1	1	1	1	1	1
	16	16	1	1	1	1	1	1
10	8	15	1	1	1	1	1	1
20			1	1	1	1	1	1
30			1	1	1	1	1	1
40			1	1	1	1	1	1
50			1	1	1	1	1	1
100			1	1	1	1	1	1
200			1	1	1	1	1	1
500			1	1	1	1	1	1

Similar to Table S2, Table S4 contains the inclusion rate between original matching results and the matching results obtained from using 1447 high-resolution spectra to search against ADAP-KDB library spectra with the preliminary search parameters listed in Table S3.

From the results in Table S4, the inclusion rates of all the preliminary parameters are close to or equal to 1. The running time in Table S3 shows that when the candidate spectra return threshold R=50, with different combinations of paired peaks in the user's spectrum (n) and ADAP-KDB library spectra (m), the average running time of searching one high-resolution user's spectrum against ADAP-KDB library spectra are close to 4 seconds. Additionally, when the n=8 and m=15, the average running time increases from 2.433 seconds to 8.826 seconds while R has increased from 10 to 500. In contrast to the running time with the original algorithm which is 6.011 seconds, the average running time is improved by about 2 seconds with the preliminary search algorithm when R is less or equal to 50.

3. Compare high-resolution spectra with low-resolution spectra

In order to understand the searching time difference between the high-resolution and low-resolution spectra using ADAP-KDB. From the query spectra that were used to search against ADAP-KDB, we checked the average number of matching peaks with close m/z values during the spectra search using 5 spectra each for the high-resolution spectra and the low-resolution spectra. The results are shown in Table S5. From Table S5, we can see the number of average matched peaks for high-resolution spectra is about 5 and around 27 for low-resolution spectra. This means when searching against the ADAP-KDB library spectra using one low-resolution spectrum, about 27 matched peaks on average for each m/z value in the query spectrum will return from the SQL query whereas the high-resolution spectrum will return about 5 matched peaks, which is 5 times less than the low-resolution spectrum. This results that the high-resolution spectra will cost less computational time in the SQL query than the low-resolution spectra when searching against ADAP-KDB and this explains why the average searching time for the low-resolution is greater than the high-resolution spectra.

Table S5: Average number of matched peaks for low-resolution and high-resolution spectra

low-res query spectra No.	Average number of matched peaks (low-res)
#1 105.0000@10.9429 MS1+	28.9069
#2 86.0000@11.2761 MS1+	33.4618
#3 147.0000@7.5933 MS1+	24.8593
#4 147.0000@7.4796 MS1+	22.4701
#5 147.0000@7.4992 MS1+	25.387
Total Average	**27.017**
**high-res query spectra No.**	**Average number of matched peaks (high-res)**
#1 299.0322@17.3740 MS1+	4.4859
#2 299.0331@17.3163 MS1+	4.5379
#3 299.0045@17.3808 MS1+	4.8392
#4 299.0254@17.2890 MS1+	4.7803
#5 73.0438@17.4420 MS1+	4.9185
Total Average	**4.919**

4. Calculation of the inclusion rate

The inclusion rate is calculated for each user spectrum by the formula

$$R = M_{prescreen} / M_{orig} \tag{1}$$

where $M_{prescreen}$ and M_{orig} are the number of library matches with the score > 0.6 returned by the ADAP-KDB spectral search with and without the prescreening respectively.

Analysis of the inclusion rates for the high resolution data shows the search algorithm returns identical results with or without the prescreening. We investigated this case further by looking at a few examples.

Figure S1. Comparison between query spectrum No.12 and matched library spectra

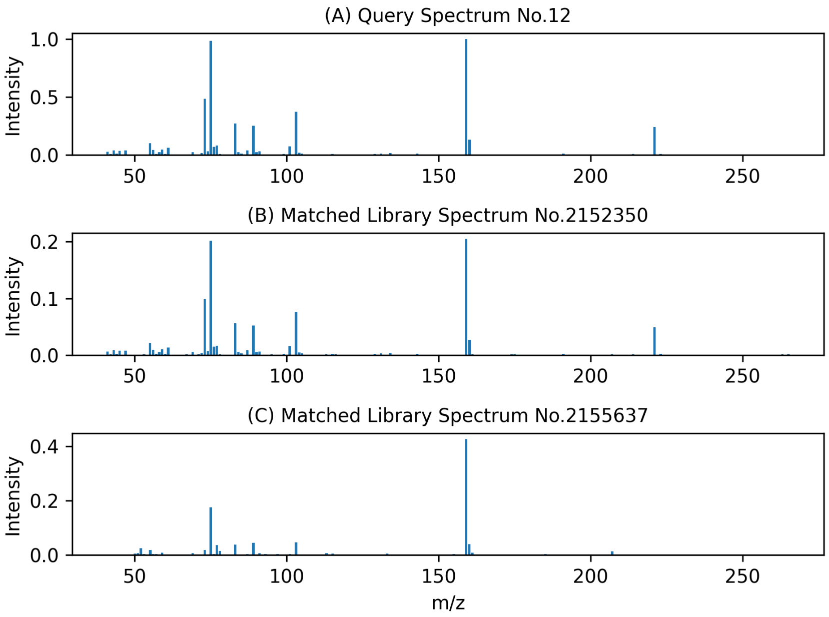

Figure S1 shows one example of the matching result using the prescreening search algorithm to search 1447 high-resolution query spectra against the ADAP-KDB library spectra when the preliminary search parameters are n=8, m=15, and R=50. Figure 1 (A) is the spectrum of the No.12 query spectrum from the 1147 high-resolution spectra data, (B) is the library spectrum No.2152350 which has a similarity score of 0.975 compared to query spectrum no.12, and (C) is the library spectrum No.2155637 which has a similarity score of 0.52 compared to query spectrum no.12. The original search algorithm results returns two matched library spectra which are library spectra No.2152350 and No.2155637 and only one of them has a score > 0.6. The same two library spectra have also been found in the result from the prescreening search algorithm. For most cases, the number of returned matched spectra for each query spectrum is around 2 in the original search algorithm. And these matched spectra can also be obtained by the prescreening search algorithm. This is why most of the inclusion rates between the preliminary search algorithm and the original algorithm are 1.

5. Prescreen Approach

Figure S2. Pre-screen Approach first step SQL pseudo-code

```sql
1  SELECT COUNT(*) AS Common, TempTable.Id FROM(
2      SELECT Id from Spectrum where ((ABS(TopMz1 - mz1) < 0.1) OR (ABS(TopMz2 - mz1) < 0.1)
3          OR (ABS(TopMz3 - mz1) < 0.1) OR (ABS(TopMz4 - mz1) < 0.1) OR (ABS(TopMz5 - mz1) < 0.1)
4          OR (ABS(TopMz6 - mz1) < 0.1) OR (ABS(TopMz7 - mz1) < 0.1) OR (ABS(TopMz8 - mz1) < 0.1))
5      UNION ALL
6      SELECT Id from Spectrum where ((ABS(TopMz1 - mz2) < 0.1) OR (ABS(TopMz2 - mz2) < 0.1)
7          OR (ABS(TopMz3 - mz2) < 0.1) OR (ABS(TopMz4 - mz2) < 0.1) OR (ABS(TopMz5 - mz2) < 0.1)
8          OR (ABS(TopMz6 - mz2) < 0.1) OR (ABS(TopMz7 - mz2) < 0.1) OR (ABS(TopMz8 - mz2) < 0.1)
9          OR (ABS(TopMz9 - mz2) < 0.1))
10     ...
11     UNION ALL
12     SELECT Id from Spectrum where ((ABS(TopMz1 - mz8) < 0.1) OR (ABS(TopMz2 - mz8) < 0.1)
13         OR (ABS(TopMz3 - mz8) < 0.1) OR (ABS(TopMz4 - mz8) < 0.1) OR (ABS(TopMz5 - mz8) < 0.1)
14         OR (ABS(TopMz6 - mz8) < 0.1) OR (ABS(TopMz7 - mz8) < 0.1) OR (ABS(TopMz8 - mz8) < 0.1)
15         OR (ABS(TopMz9 - mz8) < 0.1) OR (ABS(TopMz10 - mz8) < 0.1) OR (ABS(TopMz11 - mz8) < 0.1)
16         OR (ABS(TopMz12 - mz8) < 0.1) OR (ABS(TopMz13 - mz8) < 0.1) OR (ABS(TopMz14 - mz8) < 0.1)
17         OR (ABS(TopMz15 - mz8) < 0.1))
18 ) AS TempTable
19 JOIN Spectrum ON Spectrum.Id = TempTable.Id
20 GROUP BY Id
21 ORDER BY Common DESC
```

Figure 2 in the main paper describes the prescreen approach. Step 1 in the prescreen approach is implemented through SQL queries. Figure 4 shows a simplified version of an SQL query, which returns a table with IDs of the matched spectra for a query spectrum and the number of peaks in common between the query spectrum and each library spectrum. In the SQL query, the m/z values of the most 8 intense peaks of the query spectrum are denoted as [mz1, mz2,..., mz8] and the m/z values of the most 15 intense peaks of the library spectra are denoted as [TopMz1, TopMz2,..., TopMz15]. The m/z values of the top 15 intense peaks of all of the spectra are stored in SQL table Spectrum with columns TopMz1, TopMz2, ..., TopMz15. When this SQL query is executed, Line 2 to 4 will compare the most intense peak in the query spectrum to the top 8 most intense peaks in the library spectrum within a pre-specified m/z tolerance (e.g. 0.1) and return the library spectrum ID if one condition is satisfied. Line 5 to 9 will combine the result of comparing the second most intense peak in the query spectrum to the top 9 most intense peaks in the library spectrum within a pre-specified m/z tolerance. Line 10 to 17 will continually repeat similar comparisons and combine the results until the eighth most intense peak in the query spectrum has been compared to the top 15 most intense peaks in the library spectrum within a pre-specified m/z tolerance. Finally, Line 1 and Line 18 to 21 will generate the table with two columns which are library spectrum ID and the number of peaks in common with the query spectrum. The table will be ordered in a descendant way by the number of peaks in common with the query spectrum.
